# A rare case of disseminated erosive pustular dermatosis

**DOI:** 10.1016/j.jdcr.2025.06.007

**Published:** 2025-06-19

**Authors:** Jonathan J. Park, Joshua Prenner, Zachary Thomas, Lida Zheng, Victor L. Quan, Cuong V. Nguyen, Xiaolong A. Zhou

**Affiliations:** Department of Dermatology, Northwestern University Feinberg School of Medicine, Chicago, Illinois

**Keywords:** erosive pustular dermatosis, neutrophilic dermatosis, pyoderma gangrenosum

## Introduction

Erosive pustular dermatosis (EPD) is a rare inflammatory condition typically characterized by sterile pustules and thick crusted, hemorrhagic plaques with erosions and superficial ulcerations. EPD most commonly presents on the photodamaged scalp skin of older adults^1^ but can be seen in other sites of skin fragility such as the lower legs of individuals with venous insufficiency.^2^ The epidemiology of EPD remains unknown; a systematic review identified reports of 168 patients with EPD of the scalp and found a median age of 76 years.^3^ Fewer than 20 cases of EPD of the leg and 1 disseminated case have been reported.^4^^,^^5^ The underlying pathophysiology remains unknown, although it has clinicopathologic similarities with other neutrophilic dermatoses such as pyoderma gangrenosum (PG) and can be incited by pathergy.^6^, ^7^, ^8^ Given its rarity, EPD is often underrecognized particularly when presenting on sites beyond the scalp. We present here an exuberant case of disseminated EPD that was successfully treated with systemic steroids, oral dapsone, and infliximab.

## Case report

A 63-year-old man presented for a workup of progressive lesions on the scalp, right shoulder, back, and shins over the past year. He described thick, yellow crusts developing initially on his scalp and ulcers later on the legs after minor accidental trauma to both areas, including cuts to the head and scrapes to his legs. History was notable for actinic damage and venous stasis. Review of systems was positive for mild bilateral leg pain, pruritus, and bleeding and negative for oral or ocular discomfort, dyspnea, dysuria, abdominal pain, hematochezia, melena, fevers, chills, or unexplained weight loss. He was not up to date on age-appropriate malignancy screening. Laboratory testing was significant for mild neutrophilic leukocytosis with an absolute neutrophil count of 11,200/μL and elevated C-reactive protein (156.1 mg/L) and erythrocyte sedimentation rate (90 mm/h). Serology showed a positive antinuclear antibody panel (1:640, speckled), but extractable nuclear antigen panel, rheumatoid factor, antineutrophil cytoplasmic antibodies, and indirect immunofluorescence and enzyme-linked immunosorbent assay for epithelial antibodies were negative. Antigen or antibody testing for *Histoplasma*, *Blastomyces*, *Coccidioides*, syphilis, and HIV1/2 were negative. Contrast magnetic resonance imaging and computed tomography of the head, neck, or brain and chest, abdomen, or pelvis, respectively, did not demonstrate evidence of underlying malignancy or infectious process.

**Fig 1:**
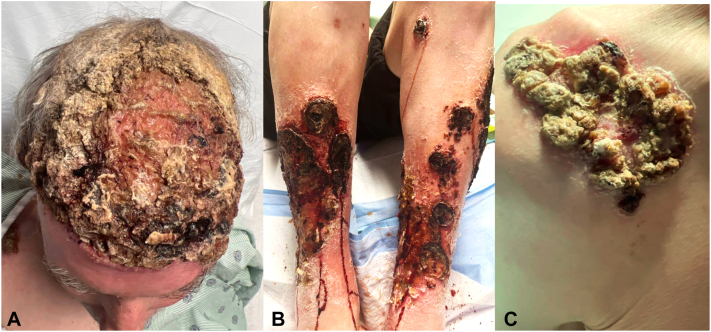
Clinical images at initial presentation. **A,** Scalp with thick, hyperkeratotic, crusted plaque involving the entire area of androgenetic alopecia. **B,** Shins with numerous hemorrhagic crusted thin ulcers surrounded by diffuse erythema. **C,** Right shoulder with thick-crusted plaque with erythematous borders.

Skin examination revealed thick yellow-crusted ulcers involving the entire alopecic scalp areas and a similar plaque on the right shoulder with erythematous borders. Both shins were characterized by diffuse erythema and induration and multiple well-demarcated thin ulcers with thick hemorrhagic crust (Fig 1, *A-C*). Multiple skin biopsies from the scalp, back, and right leg from the ulcer borders showed suppurative and granulomatous inflammation. Periodic acid-Schiff, Gram, and acid-fast stains were negative for microorganisms. There was no evidence of immune deposits on direct immunofluorescence. Tissue cultures were negative for mycobacteria and fungi and positive for mixed bacterial colonization including *Staphylococcus aureus, Staphylococcus lugdunensis, and Finegoldia magna.* Overall, the clinical presentation was consistent with disseminated EPD.

**Fig 2:**
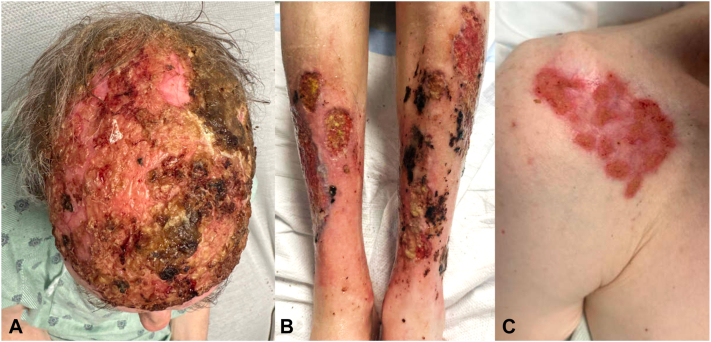
Clinical images after one week of treatment with intravenous methylprednisolone, oral dapsone, topical clobetasol, and vinegar soaks. **A,** Scalp demonstrating thinner yellow-brown crust overlying atrophic skin, visible near vertex. **B,** Shins with multiple superficial ulcers with reduced erythema, some with decreased size and increased granulation tissue. **C,** Right shoulder with well-healing thin pink erosions without crusting.

**Fig 3:**
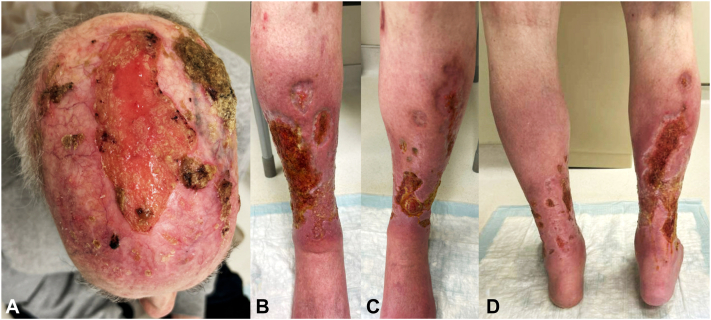
Clinical images after 3 months of treatment with infliximab and vinegar soaks. **A,** Scalp with areas of matted red-yellow crusting and healthy pink granulation tissue overlying atrophic skin. **B,** Right shin with ulcerations with yellow exudate, granulation tissue, and healing borders. **C,** Left shin with ulcerations with yellow exudate, granulation tissue, and healing borders. **D,** Posterior legs with ulcerations with yellow exudate, granulation tissue, and healing borders.

The patient was treated with intravenous methylprednisolone at 1 mg/kg/d for 1 week, dapsone 75 mg daily, topical vinegar soaks daily, and clobetasol 0.05% ointment twice daily. He showed rapid improvement within a week with sloughing of hyperkeratotic crusts, a decrease in erythema, and early re-epithelialization (Fig 2, *A-C*). He was continued on an oral prednisone taper (60 mg daily decreased by 10 mg every week) and dapsone on discharge, which was then transitioned to infliximab 5 mg/kg at weeks 0, 2, and 6 then monthly starting 2 weeks into prednisone taper. The patient is continuing treatment on monthly infliximab and daily topical vinegar soaks in the outpatient setting, with continued improvement at all sites noted after 3 months (Fig 3, *A-D*).

## Discussion

EPD is characterized by chronic, thick-crusted erosions and superficial ulcerations and is considered within the spectrum of neutrophilic dermatoses.^8^ Males are more frequently affected.^3^^,^^8^ EPD most commonly presents on the scalp in the setting of atrophy, actinic damage, androgenetic alopecia, and chronic inflammation but may occur on legs with long-standing venous insufficiency or repeated trauma.^5^ Mechanical trauma is an established precipitating factor, and like other neutrophilic dermatoses, EPD is more frequently associated with immunosuppression, myeloid disorders, and autoimmune conditions (eg, rheumatoid arthritis), although a direct pathogenic link has not been established.^6^^,^^8^ This suggests tissue damage-induced immune dysregulation with concomitant impaired wound healing as part of EPD pathogenesis. Histopathology is nonspecific and can include epidermal atrophy with amicrobial pustulation and suppurative inflammation, perifollicular granulomas, and a mixed dermal infiltrate of neutrophils, lymphocytes, and multinucleated giant cells.^6^ Similar to PG, EPD has no pathognomonic histopathologic or laboratory findings and is often a diagnosis of exclusion. Given the chronic inflammation and nonhealing wounds, EPD lesions carry an increased risk of developing secondary nonmelanoma skin cancers and scarring alopecia.^9^

In our case, the patient’s history of pathergy, suppurative inflammation on histology, and rapid improvement with steroids and dapsone therapy support a neutrophilic dermatosis etiology. The initial scalp presentation, non-cribiform scarring, and more superficial, minimally painful ulcers on atrophic skin were more suggestive of EPD than PG. However, it should be noted that some clinicians group EPD with superficial PG based on clinical presentation, pathergy, and response to similar treatment options.^10^ Clinical differential diagnoses including immunobullous disorders, fungal or bacterial infections (eg, blastomycosis-like pyoderma), cutaneous Crohn’s disease, malignancy (eg, nonmelanoma skin cancer), and vasculitis or vasculopathy were excluded after infectious, serologic, and histopathologic workup with scouting biopsies and imaging.

Reported treatments for EPD from case reports and series include topical and oral steroids, topical and systemic calcineurin inhibitors, calcipotriol, topical and oral dapsone, anti-tumor necrosis factor-alpha therapy, retinoids, zinc, oral minocycline, methotrexate, Janus kinase inhibitors, erbium YAG laser, and photodynamic therapy.^3^^,^^4^^,^^7^^,^^9^^,^^10^ Evidence supports the use of topical high-potency steroids and calcineurin inhibitors for mild cases and systemic steroids for severe cases.^6^ Importantly, potential triggers for repeated trauma need to be eliminated. Surgery and long-term use of topical steroids should be limited to prevent trauma-induced and atrophy-induced risk of disease recurrence, respectively.^3^^,^^6^^,^^9^

We present this rare case of exuberant disseminated EPD to highlight clinicopathologic features including its ability to exist beyond the scalp and effective treatment approaches.

## Conflicts of interest

None disclosed.
